# A Novel Noninvasive Method for Measuring Fatigability of the Quadriceps Muscle in Noncooperating Healthy Subjects

**DOI:** 10.1155/2015/193493

**Published:** 2015-07-21

**Authors:** Jesper B. Poulsen, Martin H. Rose, Kirsten Møller, Anders Perner, Bente R. Jensen

**Affiliations:** ^1^Department of Intensive Care, Rigshospitalet, Copenhagen University Hospital, 2100 Copenhagen OE, Denmark; ^2^Section of Integrated Physiology, Department of Nutrition, Exercise and Sport, University of Copenhagen, 2200 Copenhagen N, Denmark; ^3^Neurointensive Care Unit, Department of Neuroanaesthesiology, Rigshospitalet, Copenhagen University Hospital, 2100 Copenhagen OE, Denmark; ^4^Centre of Inflammation and Metabolism, Rigshospitalet, Copenhagen University Hospital, 2100 Copenhagen OE, Denmark

## Abstract

*Background*. Critical illness is associated with muscle weakness leading to long-term functional limitations.* Objectives*. To assess the reliability of a novel method for evaluating fatigability of the quadriceps muscle in noncooperating healthy subjects.* Methods*. On two occasions, separated by seven days, nonvoluntary isometric contractions (twitch and tetanic) of the quadriceps femoris muscle evoked by transcutaneous electrical muscle stimulation were recorded in twelve healthy adults. For tetanic contractions, the Fatigue Index (ratio of peak torque values) and the slope of the regression line of peak torque values were primary outcome measures. For twitch contractions, maximum peak torque and rise time were calculated. Relative (intraclass correlation, ICC_3.1_) and absolute (standard error of measurement, SEM) reliability were assessed and minimum detectable change was calculated using a 95% confidence interval (MDC_95%_).* Results*. The Fatigue Index (ICC_3.1_, 0.84; MDC_95%_, 0.12) and the slope of the regression line (ICC_3.1_, 0.99; MDC_95%_, 0.03) showed substantial relative and absolute reliability during the first 15 and 30 contractions, respectively.* Conclusion*. This method for assessing fatigability of the quadriceps muscle produces reliable results in healthy subjects and may provide valuable data on quantitative changes in muscle working capacity and treatment effects in patients who are incapable of producing voluntary muscle contractions.

## 1. Introduction

These years, the numbers of intensive care admissions are increasing [1] and, as critical care practice has improved over the last decades, more patients recover from intensive care [2, 3]. ICU acquired weakness [4] is a well-known sequelae associated with ICU admission leading to long-term functional limitations [5] and reduced self-perceived health-related quality of life [6].

Plausible contributors are inactivity and systemic inflammation response syndrome (SIRS), synergistically accentuating the catabolic drive leading to loss of muscle mass and neuromuscular dysfunction. Severe inflammation and increased muscle protein turnover are predominantly present during the early phase of ICU stay [7–9]. Accordingly, among ICU patients with multiorgan failure ultrasound scan of the rectus femoris muscle revealed a 16% decrease in cross-sectional area [7] and in ICU patients with septic shock muscle volume of the quadriceps muscles assessed by CT scanning images decrease by 16–20% [10]. In both of these studies, the detrimental loss of muscle mass was observed over the course of the first 7 days of ICU admission. Consequently, early interventions employed in the ICU may exhibit the greatest potential to mitigate this early and rapid loss of muscle mass and counteract their long-term effects and improve physical outcome.

Traditionally, the main means of assessing muscle function has almost exclusively been through volitional testing [11]. However, many ICU patients are, particularly in the early phase of their ICU stay temporarily, unable to cooperate (incapable of producing voluntary, consistent, and purposeful muscle activity) because of reduced level of consciousness due to critical illness in itself or due to administration of sedative medications.

There is growing awareness of the potential role of early rehabilitation [12, 13] and nonvolitional strategies (i.e., muscle stimulation) in mitigating ICU acquired weakness. Therefore, in order to predict motor deficits and need for rehabilitation and to evaluate the efficacy of potential therapies in these patients, methods which can be utilized early during the ICU admission are needed.

The aim of the present study was to develop a nonvolitional and noninvasive method to evaluate muscle fatigue in large, proximal muscle groups with clinical relevance to physical function, such as the quadriceps muscle, and assess this method in healthy subjects by measuring test-retest reliability for various fatigue parameters during tetanic and twitch contractions.

## 2. Methods

### 2.1. Participants and Assessors

Twelve healthy adult volunteers (6 men) with no previous experience with transcutaneous electrical muscle stimulation (TEMS) participated. Mean age was 22 (standard deviation, 3) years, and body mass and height were 68 (8) kg and 174 (6) cm, respectively. One subject suffered from mild asthma; no other subject had a medical history of respiratory, cardiovascular, metabolic, or neuromuscular disorders. All individuals were instructed to refrain from any strenuous activity 24 hours before each testing day.

The same two assessors (J. B. Poulsen and M. H. Rose) conducted all tests and had collectively extensive experience with transcutaneous electrical muscle stimulation and force measurements from previous research.

### 2.2. Study Design

The study was conducted in a room that was equipped for and dedicated to the study. Strict efforts were made to ensure similar testing conditions for both testing days. All measurement data was independently collected at two time points separated by 7 days, during which subject reported no change in physical or health status. Fatigue indices were measured bilaterally on the quadriceps muscle in a test-retest repeatability setup using identical protocols. Subjects were tested at the same time of day and were placed in bed in a position as outlined below. The environmental temperature was kept at 21°C. Strain gauges were calibrated and after carefully fitting electrodes for transcutaneous electrical muscle stimulation on the thigh and straps for strain gauge measurement, each subject underwent a short standardized low intensity familiarization session prior to the testing procedure to ensure potentiation of muscle tissue [14] during which electrical muscle stimulation and the study protocol were carefully explained. Left/right testing order was randomized between subjects, and the same order of testing was maintained on the second experimental day. Subjects were instructed to relax as much as possible and to avoid any voluntary contraction during the test. Testing was carried out using a protocol of two series of consecutive single-twitch contractions separated by one series of consecutive tetanic contractions. In order to reduce day-to-day variations in electrode placement on the thighs, electrodes were marked before removal, along with at least three permanent landmarks, on a transparent paper, thus serving as a “map” for a precise identification.

### 2.3. Experimental Model

The method was composed of the following three main constituents: positioning of the subject in a stabilizing device, electrical muscle stimulation, and force measurement.

**Figure 1:**
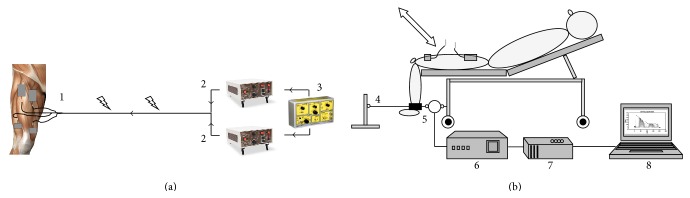
Schematic diagram of the model. (a) Electrical muscle stimulation circuit: (1) placement of self-adhesive carbon electrode pads; (2) constant current high voltage muscle stimulators; (3) train generator. (b) Force measurement circuit: (4) rigid ankle and contra strap; (5) strain gauge; (6) bridge circuit/amplifier; (7) analog-digital converter; (8) computer.

*(i) Positioning of the Subject (Figure 1)*. Subjects were semirecumbent (30°) in a hospital bed on a specially designed device consisting of a rigid padded wooden board resting on metal bar, which was attached to the metal framing of the hospital bed securing firm support of the entire thigh, uniformity of posture, joint positions, and orientation of the thigh. Isometric knee extension forces were measured with the knee joint of the stimulated leg at a 90-degree angle and the lower leg hanging vertically over the edge of the bed. This position was chosen to minimize unnecessary movement and thus reduce the risk of extubation and other complications, when applied in mechanically ventilated ICU patients.

**Figure 2:**
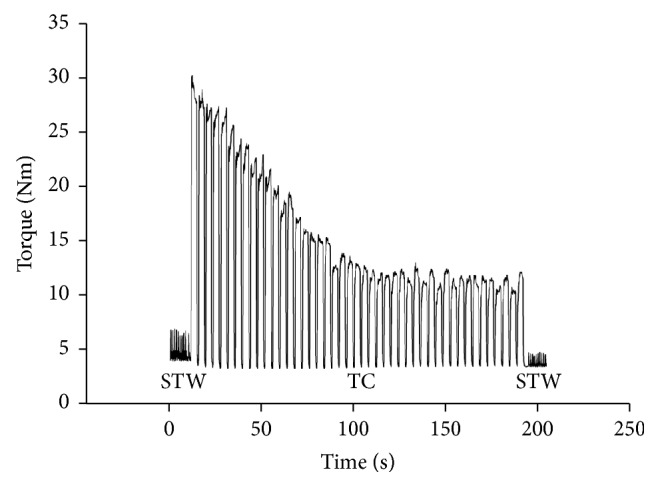
A recording of typical subject's force output during the fatigue test. An initial single-twitch series was followed by 40 tetanic contractions, after which a final single-twitch series was done. STW: single twitch; TC: tetanic contractions; Nm: newton meters.

*(ii) Electrical Muscle Stimulation*. The muscle stimulation procedure was carried out as described in a previous study [10]. Two carbon electrode pads (5 × 5 cm, Axelgaard Manufacturing Co. Ltd., PALS Platinum, Fallbrook, CA, USA) were placed over the motor point of the heads of the vastus medialis and vastus lateralis muscles [15]. Another pair of electrodes (5 × 9 cm) was placed five centimeters distal to the inguinal fold (Figure 1) [16]. The motor point was defined as the location that corresponded to the lowest possible threshold current, and the motor threshold current was defined as the lowest train stimulation current that resulted in visible muscle contraction. To optimize electrical conduction, the skin was shaved and rinsed before applying the electrodes. Initially, two constant current high voltage stimulators (model DS7A, Digitimer, Welwyn, Garden City, Hertfordshire, UK) delivered ten single stimuli twitches with biphasic square pulses at widths of 300 *μ*s. A train generator (model: Digitimer, Welwyn, Garden City, Hertfordshire, UK) was then switched on, triggering the delivery of 35 Hz current in bouts of three seconds periods separated by one second pause for a total of 40 tetanic contractions corresponding to 15 contractions per minute. In immediate succession to the tetanic contractions, a second series of 10 single-twitch stimuli ended the protocol.  Figure 2 shows an example of a recording of the force response curve.

On the first experimental day, testing current was adjusted to 75% above the motor threshold current. On the second testing day, the intensity was determined as the stimulation intensity corresponding to the same force output level (Nm) as generated by the first tetanic contraction on the first testing day, while maintaining the same ratio between the vastus medialis and the vastus lateralis stimulation intensity.

The stimulation parameters and the two-channel stimulation method were chosen in order to increase the amount of stimulated muscle mass and to reduce risk of excessive muscle damage and recruitment of sensory fibers (minimizing discomfort), thus ensuring a safe, tetanic contraction when applied to subjects, who are unable to communicate.

*(iii) Force Measurement (Figure 1)*. A height-adjustable strain gauge, placed beneath the bed, was secured to the metal framing of the bed and connected horizontally (180 degrees) to the subjects with a rigid strap placed around the ankle. To stabilize the mechanical system another rigid strap connected the ankle and a fixed metal bar, pulling lightly in the opposite direction of the strap holding the strain gauge. A bridge circuit detected changes in relative resistance in the strain gauge, and the transformed voltage changes, proportional to the torque generated by the activated muscle, were then A/D converted (Data Translation, 8  channel, USB-BNC Box), sampled at 1 kHz and subsequently stored on a computer for further offline analysis.

### 2.4. Offline Data Analysis

Full data set was achieved for all twelve participants.

*Tetanic Contractions*. To ensure attainment of a plateau level of contractions, torque values were calculated as the mean of the last second of the 3-second contraction. Torque values for each contraction were calculated and peak tetanic torque values (the highest obtained torque value within any of the below mentioned four intervals) were recorded. In order to assess the fatigability response during different time frames, the tetanic stimulation period was subdivided into four intervals:

- interval 1 (contractions 1–15, 60-second duration, the first minute);
- interval 2 (contractions 16–30, 60-second duration, the second minute);
- interval 3 (contractions 1–30, 120-second duration, the first two minutes);
- interval 4 (contractions 1–40, 160-second duration, the total period).

For each interval, the resistance to muscle fatigue was expressed as a Fatigue Index (FI), which was calculated as the ratio between the average of peak torque values from the final three contractions relative to values from the first three contractions. Recognizing that calculation of FI involves a notably condensation of data, we also calculated the slope of the regression line (SOR) for peak torque values in each interval.

*Twitch Contractions*. All data were 15 Hz low-pass filtered. For each 10-twitch series, before and after the tetanic stimulation period, peak torque and rise time (the slope between 30% and 70% of the twitch peak force, Nm/s) were calculated for the individual twitches. Both peak torque and rise time were then expressed as the average values of all twitches before and after the tetanic contraction.

### 2.5. Statistical Analysis

Due to the novelty and explorative nature of the study sample size was chosen out of convenience.

Gaussian distribution was found based on graphical plots and the Kolmogorov-Smirnov test for normality; thus, parametric methods were used to analyze data and all data were presented as means and standard deviations (SD). Paired *t*-test was used to detect potential test-retest bias.

Test-retest variation was calculated both as relative and absolute reliability. The relative reliability was expressed as intraclass correlation coefficients (ICC_3.1_) with the corresponding 95% confidence interval (CI). The following parameters were chosen during the statistical analysis of ICC_3.1_: two-way mixed effects model, consistency definition, and single measures. The classification suggested by Shrout (fair = 0.41–0.60, moderate = 0.61–0.80, and substantial > 0.81) was used to evaluate the relative reliability [17]. The absolute reliability was expressed as the standard error of measurement (SEM) with 95% CI and minimal detectable change (MDC_95%_).

Bland-Altman scatter plots were used to evaluate heteroscedasticity, that is, if the magnitude of test-retest differences was related to test-retest means. *P* values < 0.05 were considered statistically significant. All analyses were done using the Statistical Package for the Social Sciences version 18 (SPSS Inc., Chicago, IL, USA).

## 3. Results

A test session took approximately 60 minutes including setting up equipment, calibrating, patient preparation, familiarization session, and testing procedure.

Apart from redness of skin in relation to the electrodes, no adverse effects were noted. All subjects tolerated the test. However, some subjects reported moderate discomfort with the highest intensity during the initial part of the test and seemed to gradually decrease.

All subjects' force output recordings during the fatigue test showed marked signs of fatigue both during tetanic and twitch contractions on both test days (Figure 2 shows a typical recording of force output). Over the course of the full tetanic stimulation period (40 contractions), the mean ratio of the peak torque values from the final three contractions relative to values from first three contractions was 0.30 and 0.31 on test and retest day, respectively (Table 1).

No side-to-side differences were observed in motor threshold or torque measurements; therefore, mean values of left- and right-sided data were used in the analysis. No test-retest bias or heteroscedasticity were found (data not shown).

Absolute values and differences of motor threshold, tetanic contractions (peak torque, FI, and SOR), and twitch contractions (peak torque and rise time) and corresponding reliability data (ICC_3.1_, SEM, and MDC_95%_) are presented in Table 1.

- The motor threshold value of vastus medialis was significantly lower than that of vastus lateralis (Table 1).
- For tetanic contractions, the very first peak torque value, which was used to calibrate stimulation intensity between test and retest day, demonstrated substantial relative reliability (ICC_3.1_ = 0.97). Fatigue Index during interval 1 showed substantial relative reliability (ICC_3.1_ = 0.84) and MDC_95%_ = 0.12. The regression line of the peak torque values (SOR) during the first two minutes (interval 3, contractions 1–30) showed both substantial relative and absolute reliability (Table 1).
- For twitch contraction, no parameter showed both substantial relative and absolute reliability (Table 1).

## 4. Discussion

In the present study, we present a new noninvasive method for the evaluation of muscle fatigue resistance of the quadriceps muscle in healthy subjects. Although there were considerable variations in peak torque, FI, and SOR between subjects, the intraindividual reproducibility of these measurements were highly consistent with a substantial reliability of motor threshold and tetanic contraction. In contrast, the reliability of data originating from twitch contractions values was less reproducible.

The quadriceps is the primary locomotor muscle for activities of daily living (ADL) [18]. Accordingly, quadriceps weakness has been associated with decreased ADL performance, increased incidence of falls [19, 20], and mortality in elderly subject [21]. Thigh muscles have shown to be very sensitive to muscle atrophy particularly acquired during the early phase of the ICU stay [7]; they are subject to a larger degree of muscle atrophy than muscles of the upper extremities after bed rest [22] and one year after ICU admission the fatigue resistance is still reduced compared to an age and gender matched control group [23]. Therefore, we find that changes in quadriceps fatigability may be an appropriate surrogate marker for important functional outcomes, such as ADL and physical function. On that basis, we designed the present method which compare differences in fatigue response in the quadriceps muscle. The most important novelty of our method is the direct comparability of performed muscle work/force output by calibrating values of initial torque response on each testing day. The method may therefore be more robust to several potential biases which may influence tissue conductivity, thus enabling a low bias functional assessment of muscle fatigue, at different time points, that is, before and after an intervention.

During consecutive TEMS sessions, the stimulus threshold has been shown to increase with increased severity of illness in ICU patients [10], sepsis, and use of vasopressors [24]. This is most likely due to changes in tissue and sarcolemmal conductivity. Muscle force response to electrical stimulation is influenced by a variety of intrinsic (patient-dependent) factors, such as body mass index [25], tissue blood flow, subcutaneous oedema [26], inflammatory, metabolic and electrolyte changes [27, 28], and extrinsic factors such as electrode placement [29, 30]. In critically ill patients, these intrinsic parameters are subject to a large day-to-day variability, which may render the clinical muscle response to TEMS with a predefined fixed* stimulation intensity* less consistent. In order to establish stable testing condition, healthy volunteers were chosen and by calibrating baseline torque on each testing days we aimed at obtaining a reproducible and comparable muscle torque during the first tetanic contraction regardless of the stimulation intensity level and electrode placement. Our data show that a predefined fixed* torque response* is accurately reproduced (ICC_3.1_ = 0.97), which suggests that, compared to a fixed stimulation intensity level on both days, our method may potentially circumvent fluctuations of parameters that influence tissue conductivity.

Another novelty of our method is the two-channel stimulation technique. Our data showed threshold values of vastus lateralis that was up to 50% higher than those of vastus medialis. This difference in muscle activation sensitivity may cause either understimulation of vastus lateralis or overstimulation of vastus medialis when a one channel stimulation protocol is used. Consequently, using a two-channel stimulation technique may help better quadriceps muscle activation and patient comfort and thus may be preferable over using one channel stimulation only.

Compared to tetanic contractions, we observed a high degree of test-retest variability in torque measurements during twitch contractions. Some baseline drift due to the lack of rigidity in the mechanical setup may in part explain the lower reliability of twitch contractions. Compared to tetanic contractions, we observed a high degree of test-retest variability in torque measurements during twitch contractions. Some baseline drift due to the lack of rigidity in the mechanical setup may in part explain the lower reliability of twitch contractions.

McDonnell et al. [31] and Snyder-Mackler et al. [32] both used electrical muscle stimulation to assess quadriceps fatigability. The methods of both studies required cooperating subjects, as baseline comparability was ensured by stimulating to a specific percentage of maximum voluntary contraction (%MVC), 60 and 20% MVC, respectively. However, the reported decline in torque response (30–50% of first recorded value) and the reliability values (ICC 0.83) are comparable to our results.

Eikermann and colleagues [33] measured nonvoluntary force in the adductor pollicis muscle using peripheral nerve stimulation of the ulnar nerve and found a likely myopathy- and/or axonal neuropathy-induced peripheral muscle weakness in ICU patients (*n* = 13), without evidence for an increased fatigability. The response of a specific skeletal muscle to immobilization and inflammation will, among other factors, depend on its muscle fiber composition. Thus, the effect of immobilization and inflammation on contractile properties (i.e., fatigue) may vary between different muscle groups, which may contribute to muscle group and task-specific muscle fatigability. This is supported by Man et al. who found significant weakness in the quadriceps muscle despite a preserved strength in the adductor pollicis muscle of COPD. Consequently, direct comparison between the present study and other nonvoluntary protocols may be hampered due to the specific protocol used (i.e., choice of muscle group and stimulation method).

There are several important limitations to the present study. First, a major consideration in the present study is the exploratory design. Our results are critically dependent on the selected experimental settings, such as a convenient sample size and usage of health volunteers. Even though the use of health volunteers provides stable test conditions, both critical illness and age may influence muscle function and composition [34]. Our results can therefore not be extrapolated to an ICU population and cross-validation in this population needs to be confirmed. These factors both limit the overall generalizability and strengths of our conclusions, which must be interpreted in this context.

Furthermore, the torque response during the fatigue test depends on the fiber type distribution of the stimulated muscle [35]. This reduces the generalizability of our results to other muscle groups with a different muscle fiber composition. Furthermore, despite reports of a high correlation between electrical and voluntary response to muscle fatigue protocols [36, 37], muscle tissue adapts differently to fatigue during voluntary exercise than during electrical stimulation (recruitment pattern, firing frequencies, etc.) [38]. These differences in adaptive strategies and the magnitude of change in muscle fatigue that will represent a clinically meaningful change for the patient should be considered when interpreting the present data in the context of physiological and clinical outcomes. Given the importance of the quadriceps for most activities of daily living, a reliable method for measuring these characteristic might prove valuable for prognostication and trials to prevent ICU acquired weakness, that is, by neuromuscular electrical stimulation [39, 40]. Also, due to the moderate complexity of the experimental setup, sensitivity of the equipment, and the offline data measurements, and to ensure consistent measurements and high quality data, a basic knowledge of muscle force measurements and transcutaneous electrical muscle stimulation equipment is required. This may limit the application of present method to research only, but may be the first step in the challenge to find a test which can be performed at the bedside in clinical practice.

In conclusion, the present noninvasive, nonvoluntary method for assessing fatigability of the quadriceps muscle produces reliable results in healthy subjects. It may have the potential to provide valuable data on quantitative changes in muscle working capacity and treatment effects in critically ill patients during the early phase of ICU admission.

## Figures and Tables

**Table 1:** Descriptive and reliability data.

Variable	Test visit	Retest visit	Difference	ICC_3.1_ (95% CI)	SEM (95% CI)	MDC_95%_
Motor threshold (mA)						
Vastus medialis	16.32 ± 4.53	17.68 ± 4.45	1.36 ± 1.85	0.92 (0.72–0.98)	1.29 (−1.24–3.82)	3.57
Vastus lateralis	18.86 ± 2.96	19.86 ± 1.63	1.00 ± 1.41	0.64 (0.11–0.89)	1.02 (−0.98–3.03)	2.84
Tetanic contractions						
Peak torque (Nm)	27.03 ± 14.76	29.28 ± 17.09	2.25 ± 3.90	0.97 (0.89–0.99)	2.70 (−2.59–7.99)	7.48
Fatigue Index						
Interval 1 (contract. 1–15)	0.57 ± 0.11	0.59 ± 0.11	0.02 ± 0.06	0.84 (0.51–0.95)	0.04 (−0.04–0.12)	0.12
Interval 2 (contract. 16–30)	0.69 ± 0.08	0.70 ± 0.09	0.01 ± 0.09	0.51 (0.09–0.84)	0.06 (−0.06–0.18)	0.17
Interval 3 (contract. 1–30)	0.33 ± 0.08	0.35 ± 0.10	0.02 ± 0.05	0.82 (0.46–0.95)	0.04 (−0.04–0.11)	0.10
Interval 4 (contract. 1–40)	0.30 ± 0.09	0.31 ± 0.10	0.01 ± 0.07	0.71 (0.22–0.91)	0.05 (−0.05–0.15)	0.14
Slope of regression line (Nm/s)						
Interval 1	−0.86 ± 0.20	−0.82 ± 0.23	0.04 ± 0.14	0.77 (0.36–0.93)	0.10 (−0.10–0.30)	0.28
Interval 2	−0.25 ± 0.08	−0.23 ± 0.07	0.02 ± 0.08	0.52 (−0.08–0.84)	0.05 (−0.05–0.16)	0.15
Interval 3	−0.20 ± 0.12	−0.20 ± 0.12	0.00 ± 0.02	0.99 (0.96–1.00)	0.01 (−0.01–0.04)	0.03
Interval 4	−0.19 ± 0.11	−0.14 ± 0.09	0.05 ± 0.05	0.85 (0.54–0.96)	0.04 (−0.04–0.11)	0.11
Twitch contractions						
Initial twitch series						
Peak torque (Nm)	2.63 ± 1.32	2.95 ± 1.33	0.32 ± 1.12	0.64 (0.11–0.89)	0.78 (−0.75–2.30)	2.16
Rise time (Nm/s)	40.60 ± 17.94	35.52 ± 15.49	−5.08 ± 9.45	0.84 (0.51–0.96)	6.61 (−6.34–19.55)	18.31
Final twitch series						
Peak torque (Nm)	1.23 ± 0.65	1.33 ± 0.60	0.10 ± 0.33	0.87 (0.58–0.96)	0.23 (−0.22–0.67)	0.62
Rise time (Nm/s)	20.82 ± 11.50	17.78 ± 8.92	−3.04 ± 10.65	NS		

Data are expressed as means ± standard deviations.

ICC_3.1_: intraclass correlation coefficients; CI: confidence interval; SEM: standard error of measurement; MDC_95%_: minimal detectable change; mA: milliamperes; Nm: newton meters; NS: not significant.
